# Efficacy and Safety Evidence Supporting Cancer Drug Approvals in Switzerland (2001–2020): A Meta-Analysis of Pivotal Randomised Controlled Trials

**DOI:** 10.1016/j.lanepe.2026.101710

**Published:** 2026-05-14

**Authors:** Parisa Rahimzadeh, Qiyu Li, Leonie Rudofsky, Simon Dalla Torre di Sanguinetto, Nicola Miglino, Jeanette Bachir, Nicole Bodmer, Arūnas Girčys, Mario Iovino, Stephanie Juritz, Kathrin Espinoza, Bettina Maier, Elisa Zaninotto, Matea Zosso-Pavic, Anita Wolfer, Ulrich-Peter Rohr, Andreas Wicki

**Affiliations:** a Faculty of Medicine, University of Zurich, Zurich, Switzerland; b Division Clinical Assessment, Authorisation Sector, Swiss Agency for Therapeutic Products (Swissmedic), Bern, Switzerland; c Division Regulatory Operations and Development, Swiss Agency for Therapeutic Products (Swissmedic), Bern, Switzerland; d Human Medicines Expert Committee, Swiss Agency for Therapeutic Products (Swissmedic), Bern, Switzerland; e Department of Oncology, University Hospital Geneva, Geneva, Switzerland; f Department of Medical Oncology and Haematology, University Hospital Zurich, Zurich, Switzerland

**Keywords:** Antineoplastic agents, Drug approval, Treatment outcome, Meta-analysis, Switzerland

## Abstract

**Background:**

Randomised controlled trials (RCTs) with overall survival (OS) as the primary endpoint remain the gold standard for evaluating the efficacy and safety of new cancer therapies. However, increasing reliance on early or intermediate endpoints such as progression- or recurrence-related time-to-event endpoints (PRTTE) including progression-free survival (PFS) and disease-free survival (DFS) has led to concerns about the strength and clinical relevance of the evidence at the time of approval. We aimed to systematically evaluate the evidence supporting new cancer drugs approved by Swissmedic, focusing on efficacy and safety measures and differences in treatment effects between OS and PRTTE, mainly PFS and DFS, across different cancer types, drug classes, and trial characteristics.

**Methods:**

We identified pivotal clinical trials from the Swissmedic database supporting new active substances and indication extensions between Jan 1, 2001, and Dec 31, 2020. We included RCTs and excluded single-arm, non-inferiority studies, and those trials that did not report on the prespecified outcomes of interest (OS, PRTTE, or response rate). Meta-analysis using random-effects models was performed to pool hazard ratios (HRs) for OS and PRTTE, odds ratios (ORs) for response rates, and risk ratios (RRs) for serious adverse events (SAEs).

**Findings:**

We identified 241 RCTs supporting approvals of 102 cancer drugs. PRTTE endpoints, mainly PFS, were the primary endpoint in 69.2% (167/241) of trials, while OS was the primary endpoint in 30.2% (73/241). Across 194 RCTs reporting OS, PRTTE and SAEs, the newly approved treatment reduced the risk of death by 24% (HR 0.76; 95% CI 0.74–0.77) and risk of the progression or recurrence by 45% (HR 0.55; 95% CI 0.52–0.58) compared with the control arm, with an increased relative risk of SAEs by 26% (RR 1.26; 95% CI 1.21–1.32). Absolute median survival gains were modest (OS 2.42 months; PRTTE (PFS) 3.47 months). In the palliative setting, differences in treatment effect between OS and PRTTE (PFS) varied across solid cancer types and drug classes. Among tumour types, differences between OS and PRTTE were more pronounced in ovarian cancer (OS HR 0.87 vs. PRTTE HR 0.55), whereas in head and neck cancer the estimates were similar (OS HR 0.72 vs. PRTTE HR 0.70). Among drug classes, targeted agents showed more pronounced differences (OS HR 0.74 vs. PRTTE HR 0.48), whereas immune checkpoint inhibitors showed similar effects (OS HR 0.71 vs. PRTTE HR 0.72).

**Interpretation:**

The cancer drugs approved by Swissmedic were associated with treatment benefits and modest median survival gains, accompanied by an increased risk of SAEs at the time of approval. The reliance on early endpoints, limited availability of long-term OS data, and the observed differences between PRTTE, mainly PFS/DFS, and OS highlight challenges in interpreting evidence at the time of approval. These findings suggest that treatment effects should be interpreted in the specific clinical and biological context, with enhanced post-approval OS data collection to support the regulatory and clinical assessments.

**Funding:**

University of Zurich.

**Research in context**

**Evidence before this study**

Over the past two decades, the number of cancer drug approvals has increased substantially, reflecting advances in oncology drug development. While overall survival (OS) remains the most robust outcome measure, there is a growing reliance on early or intermediate endpoints such as progression- or recurrence-related time-to-event endpoints (PRTTE), including progression-free survival (PFS) and disease-free survival (DFS), in trials supporting drug approvals. Consequently, there is an ongoing debate on the completeness, robustness, and clinical relevance of the evidence available at the time of approval.

We searched PubMed for peer-reviewed, original studies published from database inception to March 1, 2026, without language restrictions, using the combination of search terms related to cancer drug approvals and clinical outcomes. The search strategy included terms such as (“cancer” OR “antineoplastic agents”) AND (“drug approval” OR “regulatory approval”) AND (“overall survival” OR “progression-free survival” OR “disease-free survival”). Our search identified several evaluations of the evidence supporting approvals particularly by the U.S. Food and Drug Administration (FDA). Most evaluations have focused on measures of efficacy without parallel assessment of safety. In addition, a subset of studies acknowledged discrepancies between OS and PRTTE, however, comprehensive assessments comparing the treatment effects between OS and PRTTE across cancer types, drug classes, and trial characteristics remain limited.

**Added value of this study**

We systematically evaluated the evidence of clinical efficacy and safety supporting cancer drugs authorised in Switzerland between 2001 and 2020. This meta-analysis included 241 randomised controlled trials (RCTs) supporting approvals of new active substances and indication extensions. PRTTE, mainly PFS, was continuously used more frequently than OS as the primary endpoint in pivotal trials. Approved cancer drugs were associated with a considerable reduction in the risk of death and progression or recurrence, but with modest absolute survival gains and were accompanied by an increase in SAEs, compared with the control arm. However, the differences in treatment effects between OS and PRTTE (PFS) varied across solid cancer types and drug classes in the palliative setting with close alignment in some settings such as head and neck cancer and among immune checkpoint inhibitors, and greater divergence in others, including ovarian cancer and targeted agents.

**Implications of all the available evidence**

The magnitude of treatment effects on OS and PRTTE (PFS/DFS) should be interpreted with caution, as concordance between these endpoints may vary by cancer type and drug class. These differences highlight the need to evaluate early or intermediate endpoints within their specific clinical and biological context. From a regulatory perspective, the limited availability of OS as a primary endpoint and the reliance on early or intermediate endpoints highlight the ongoing challenges in drug assessment. Ensuring timely access to meaningful OS data, either at initial approval or through robust post-approval evidence requirements, remains essential for informed decision-making.

## Introduction

Despite major improvements in survival across various cancer types in the last two decades, cancer continues to be one of the leading causes of death worldwide.^1^ Between 2001 and 2020, the number of cancer drug approvals increased significantly, reflecting scientific innovation and an increased pace of cancer drug development.^2^

From a regulatory perspective, a new drug can be approved if it demonstrates a positive benefit-risk profile, meaning its benefits and risks are well characterised and the benefits outweigh the risks for the intended population.^3^ Randomised controlled trials (RCTs) are considered the gold standard for primarily assessing the efficacy and safety of new cancer treatments.^4^ Overall survival (OS) is considered the most robust and clinically relevant endpoint.^5^ However, its use as a primary endpoint is limited by the need for longer follow-up and by factors such as crossover and post progression therapies, which may reduce observable differences in OS between study arms and make the attribution of survival benefit to the initial intervention more challenging to assess.^6^, ^7^, ^8^ As a result, early or intermediate endpoints such as progression- or recurrence-related time to event endpoints (PRTTE), including progression-free survival (PFS) and disease-free survival (DFS), as well as response-based endpoints such as objective response rate (ORR) are often used.^9^^,^^10^ PRTTE endpoints can shorten study duration and costs, thereby facilitating a timely assessment of the efficacy of new therapies, which may lead to faster approvals.^11^ However, these measures may be weak predictors of actual OS.^12^ With the growing reliance on early or intermediate endpoints, concerns have been raised regarding the completeness, medical relevance, and quality of the clinical evidence supporting oncology drug approvals. Despite systematic evaluations of the evidence supporting cancer drug approvals by the U.S. Food and Drug Administration (FDA) and the European Medicines Agency (EMA),^13^, ^14^, ^15^, ^16^, ^17^, ^18^, ^19^ comprehensive assessments of the efficacy and, in particular, safety evidence at approval, as well as differences in treatment effects between OS and PRTTE, remain limited.

We aimed to systematically evaluate clinical efficacy and safety evidence supporting cancer drugs authorised by Swissmedic between 2001 and 2020. Our study included most commonly used and clinically relevant endpoints reported in pivotal trials at the time of approval, and compared treatment effects across cancer types, drug classes, and trial characteristics.

## Methods

### Search strategy and selection criteria

We retrospectively identified the pivotal clinical trials supporting cancer drug approvals in Switzerland from Jan 1, 2001, to Dec 31, 2020, using the internal Swissmedic database. Pivotal RCTs supporting the approval of new active substances and indication extensions were included. We excluded single-arm studies, trials with non-inferiority design, those supporting non-systemic, biosimilar, and supportive care drugs, and the studies not reporting results for any of the prespecified outcomes of interest (OS, PRTTE, or response rate).

### Procedure

Data were extracted from Swissmedic clinical assessment reports and submitted dossiers from the applicants at the time of approval. Data extraction was performed by P.R. using a predefined framework. Data extraction, data quality evaluation and data cleaning were conducted between December 2022 and December 2024.

For each RCT, we collected information on drug class, defined by the mechanism of action, cancer type and approved indication label, trial characteristics (phase, blinding, trial comparator, treatment setting, line of therapy, (co)primary endpoints, type I error-controlled endpoints, crossover), and outcome data.

We classified the outcome data as 1. OS and PRTTE (including PFS, time to progression (TTP), metastasis-free survival (MFS), DFS, relapse-free survival (RFS), and event-free survival (EFS)), 2. Response rate, and 3. Safety outcomes, including serious adverse events (SAEs), defined according to ICH E2A^20^ and irrespective of treatment attribution, and adverse events (AEs) grade 3–4 and 5, defined according to the Common Terminology Criteria for Adverse Events (CTC-AE). As the included PRTTE endpoints consisted predominantly of PFS in the palliative setting and DFS in the curative setting, these endpoints are collectively referred to as PFS/DFS. In analyses restricted to the palliative setting, the term PFS is used. Data were extracted according to the approved indication label and the most updated results available at the time of approval. Subgroup-specific results were used when approvals were restricted to subpopulations; otherwise, intention-to-treat data were used.

For time-to-event endpoints, we extracted sample size, number of events per arm, hazard ratios (HRs) with 95% confidence intervals (CIs), and median survival times, when available, with 95% CIs in both arms. Analyses were classified as interim or primary based on whether the prespecified number of events recorded in the statistical analysis plan had been reached at approval.

### Data validation and quality control

To ensure data quality, a preplanned, structured validation process was implemented. Systematic review of all entered data from randomly selected applications was performed by independent reviewers who are clinical assessors from Swissmedic. This systematic review was conducted in iterative rounds, with discrepancies resolved by consensus or, when necessary, adjudicated by senior oncologists, improving the consistency of data extraction. After several rounds, covering 30% of indication extensions and 20% of new active substance applications, discrepancies were close to zero; further quality checks were therefore discontinued, and the data were considered sufficiently robust.

### Statistical analysis

We descriptively summarised the trial characteristics. Trends in the use of different primary endpoints were summarised across four consecutive five-year intervals.

Odds ratios (ORs) were calculated for response rate and risk ratios (RRs) and risk differences (RDs) for safety outcomes. A continuity correction of 0.5 was applied to account for cases of zero events. To evaluate and compare efficacy and safety outcomes across trials, we identified trials that reported OS, PRTTE, and SAEs data at approval, creating a matched dataset. Safety was primarily assessed using SAEs, as these were most consistently reported, whereas AEs were reported heterogeneously across trials (e.g., grade 3–4 vs. grade 3–4–5).

Effect estimates were pooled using random-effects models for meta-analysis.^21^ Statistical heterogeneity was assessed with the *I*^2^ statistics. Meta-analyses were conducted overall and stratified by cancer type (solid tumours vs. haematological neoplasms). Absolute risks for SAEs and AEs were meta-analysed for the experimental and control arms.^22^ Absolute median OS and PRTTE gains (treatment minus control) were pooled using Wald approximation-based approach^23^ implemented in the R package metamedian.^24^

Subgroup analyses were performed for solid tumours by treatment settings. Trials in the palliative setting were further stratified by cancer type, drug class, and trial characteristics. Meta-analyses were conducted for subgroups with at least four trials. In addition, we conducted exploratory analyses based on the line of therapy among solid tumour trials in the palliative setting, where sufficient trial numbers were available.

Univariate meta-regression analyses were used to explore the association between study-level characteristics and treatment effects. Each covariate was examined individually using a mixed-effects meta-regression model with restricted maximum likelihood (REML) to estimate between-study variance. The Knapp-Hartung^25^ method was used to adjust the estimated standard errors for the estimated coefficients to account for the uncertainty in the estimate of the amount of residual heterogeneity. The 95% CIs of the regression coefficients were obtained from t statistics and the omnibus test to investigate the association between the covariate and outcome was based on the F test. R packages meta (version 8.2-1) and metafor (version 4.8-0) were used to perform all the meta-analyses.^26^^,^^27^ A two-sided p-value of less than 0.05 was considered statistically significant. Statistical tests were not adjusted for multiple testing. Analyses were conducted using R software (version 4.5.1; R Foundation for Statistical Computing) and RStudio (version 202 5.5.1.513; RStudio, PBC).

### Ethics statement

The analysed outcome data did not involve the collection or use of identifiable individual patient data and therefore do not fall under the Swiss Human Research Act; accordingly, approval from an ethics committee was not required.

### Reporting

This study was conducted and reported in accordance with the Preferred Reporting Items for Systematic Reviews and Meta-Analyses (PRISMA) 2020 guidelines, where applicable.

### Role of the funding source

Prof. Andreas Wicki as representative of the University of Zurich was involved in the study concept and design, interpretation of the data, writing and editing of the manuscript, and in the decision to submit the paper for publication.

## Results

**Fig. 1:**
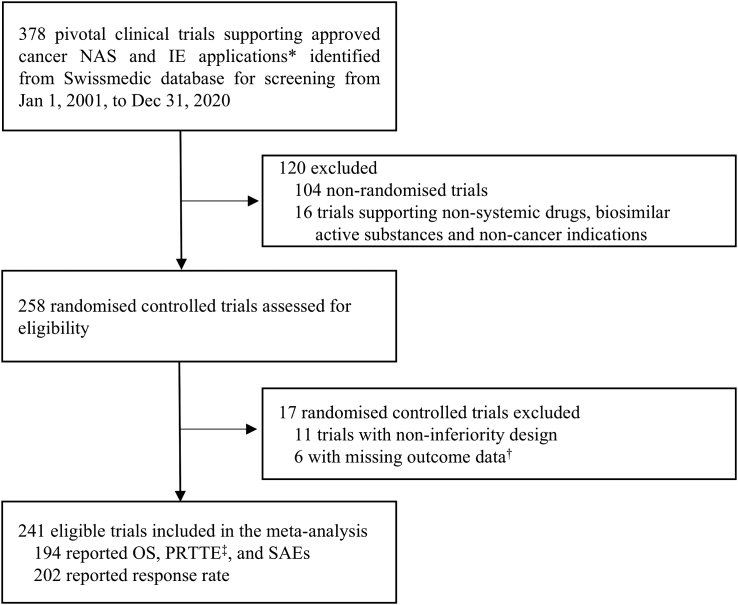
**Flowchart of study selection.** NAS = new active substance. IE = indication extension. OS = overall survival. PRTTE = progression- or recurrence-related time-to-event endpoints. SAEs = serious adverse events. ∗Applications for antineoplastic agents L01-04. ^†^Six haematology trials were excluded because none reported the outcomes prespecified for this meta-analysis (OS, PRTTE, or response rate). ^‡^PRTTE consisting predominantly of progression-free survival and disease-free survival, with limited number of time to progression, relapse-free survival, event-free survival, and metastasis-free survival.

We identified a total of 378 pivotal clinical trials supporting cancer drug approvals in Switzerland between Jan 1, 2001, and Dec 31, 2020. After full review, 241 eligible RCTs fulfilled the inclusion criteria and were included in the final analysis (Fig. 1). These 241 clinical trials supported the approval of new active substances and indication extensions for 102 distinct drugs (Appendix p 1). Most RCTs (171/241 [71%]) evaluated treatments for solid tumours and 97/241 (40.2%) investigated targeted agents (Table 1). Table 1 summarises the main characteristics of the pivotal trials.

**Table 1:** Characteristics of pivotal randomised controlled trials supporting cancer drug approvals in Switzerland, 2001–2020.

	Number of trials
Total	241 (100%)
Cancer type	
Solid tumours	171 (71%)
Breast	34 (19.9%)
Lung	28 (16.4%)
Colorectal	20 (11.7%)
Melanoma	14 (8.2%)
Renal	12 (7%)
Ovarian	12 (7%)
Sarcoma/GIST	8 (4.7%)
Gastroesophageal	7 (4.1%)
Head and neck	7 (4.1%)
Prostate	6 (3.5%)
Liver	5 (2.9%)
Other^a^	18 (10.5%)
Haematological neoplasms	70 (29%)
Leukaemia	31 (44.3%)
Multiple myeloma	20 (28.6%)
Lymphoma	13 (18.6%)
Other^b^	6 (8.6%)
Treatment type	
Monotherapy	112 (46.5%)
Combination	129 (53.5%)
Drug class^d^	
Targeted agent	97 (40.2%)
Monoclonal antibody	58 (24.1%)
Immune checkpoint inhibitor	35 (14.5%)
Cytotoxic agent	29 (12%)
Antibody-drug conjugate	8 (3.3%)
Endocrine therapy	5 (2.1%)
Other^c^	9 (3.7%)
Indication type	
New active substance	77 (32%)
Indication extension	164 (68%)
Treatment setting	
Curative setting	24 (10%)
Palliative maintenance setting	16 (6.6%)
Palliative setting	201 (83.4%)
Treatment line (palliative setting)	
First line	103 (51.2%)
Second and/or further line	88 (43.8%)
Any line	10 (5%)
Pivotal trial phase	
I/II	2 (0.8%)
II	16 (6.6%)
II/III	2 (0.8%)
III	221 (91.7%)
Type of blinding	
Open label	146 (60.6%)
Double-blinded	95 (39%)
Control arm	
Active control	185 (76.8%)
Placebo control	49 (20.3%)
No treatment/observation	7 (2.9%)
Primary endpoints^e^	
Progression or recurrence related time-to-event	167 (69.2%)
Progression-free survival	135 (80.6%)
Disease-free survival	14 (8.4%)
Event-free survival	3 (1.8%)
Recurrence-free survival	4 (2.4%)
Time-to-progression	9 (5.4%)
Metastasis-free survival	2 (1.2%)
Overall survival	73 (30.2%)
Response rate	28 (11.6%)

Data are n (%). GIST = Gastrointestinal Stromal Tumour.

a Thyroid, plural, pancreas, neuroendocrine, brain, tuberous sclerosis complex.

b Waldenström macroglobulinaemia, Myelodysplastic Syndrome, Multicentric Castleman’s disease, Polycythaemia Vera, Myelodysplastic/myeloproliferative syndrome.

c Immunomodulatory agents, oncolytic virus therapies, macrophage activator, histone deacetylase inhibitor.

d Drug classes and the corresponding subclasses are listed in the Appendix p 1.

e In some clinical trials, multiple primary endpoints (co-primary) were used, causing the total number to exceed 241 and percentage to exceed 100%.

**Fig. 2:**
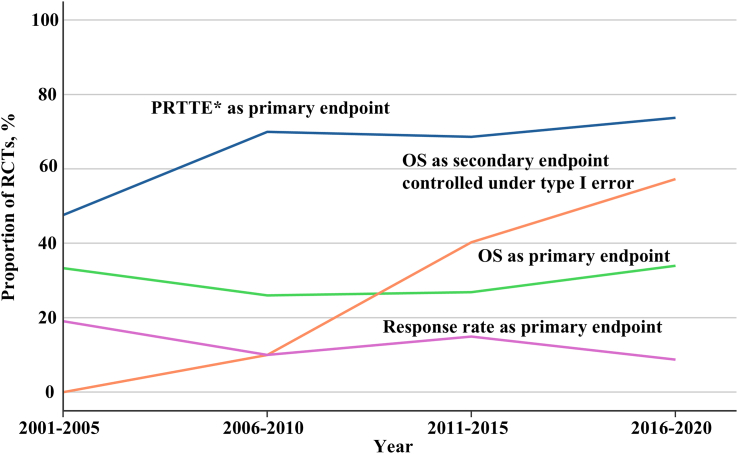
**Temporal trends in primary endpoints of pivotal randomised controlled trials supporting cancer drug approvals in Switzerland, 2001–2020.** RCT = randomised controlled trials. PRTTE = progression- or recurrence-related time-to-event endpoints. OS = overall survival. The figure shows the proportions of primary endpoints and overall survival as a controlled key secondary endpoint over time. In some clinical trials, multiple primary endpoints (co-primary) were used, causing the total percentage to exceed 100%. ∗PRTTE consisting predominantly of progression-free survival and disease-free survival, with limited number of time to progression, relapse-free survival, event-free survival, and metastasis-free survival.

The proportion of trials with PRTTE as primary endpoint was higher (167/241 [69.2%]) than those with OS as primary endpoint (73/241 [30.2%]). From 2001 to 2020, PRTTE, mainly PFS, were consistently selected more frequently as primary endpoints than OS, reaching 73.8% in 2016–2020 (Fig. 2). OS as primary endpoint remained stable over time, ranging from 26% to 34%. In parallel, the percentage of trials including OS as a key secondary endpoint (controlled under type I error) increased considerably, from 0% in 2001–2005 to 57.3% in 2016–2020. Among trials with OS as the primary endpoint, 32 (43.8%) reported an interim analysis, compared with 82 (49.1%) with PRTTE as the primary endpoint.

Of 241 trials, 194 reported OS, PRTTE, and SAEs (matched dataset). The new approved cancer drugs reduced the risk of death by 24% compared with the control arm (OS HR 0.76; 95% CI 0.74–0.77; I^2^= 24.5%) (Table 2). The risk of disease progression or recurrence was reduced by 45% (PRTTE (PFS/DFS) HR 0.55; 95% CI 0.52–0.58; I^2^= 86.6%).

**Table 2:** Treatment outcomes for overall survival, progression- or recurrence-related time to event endpoints, and serious adverse events (matched dataset) in randomised controlled trials supporting cancer drug approvals in Switzerland, 2001–2020.

	Overall Survival					Progression- or recurrence-related time-to-event^a^				Serious Adverse Events			
	No. of trials	Events/total population experimental arm	Events/total population control arm	HR (95% CI)	P-value^b^	Events/total population experimental arm	Events/total population control arm	HR (95% CI)	P-value^b^	Events/total population^c^ experimental arm	Events/total population^c^ control arm	RR^e^ (95% CI)	P-value^b^
A-ll trials	194	21,203/61,261	20,118/52,523	0.76 (0.74–0.77)		30,596/60,569	30,622/51,879	0.55 (0.52–0.58)		22,004/63,156	15,267/54,503	1.26 (1.21–1.32)	
Cancer type					0.64				0.078				0.36
Solid tumours	142	18,119/48,808	16,750/40,590	0.76 (0.74–0.78)		25,505/48,214	24,278/40,041	0.57 (0.54–0.60)		16,164/50,711	10,815/42,764	1.28 (1.21–1.36)	
Haematological neoplasms	52	3084/12,453	3368/11,933	0.75 (0.70–0.79)		5091/12,355	6344/11,838	0.50 (0.44–0.57)		5840/12,445	4452/11,739	1.22 (1.12–1.32)	
Solid tumours, Treatment setting					0.57				0.037				<0.0001
Curative	13	958/10,159	1134/9890	0.74 (0.68–0.82)		1835/10,159	2424/9890	0.57 (0.46–0.71)		2483/10,335	1706/10,632	1.70 (1.29–2.25)	
Palliative maintenance	12	1222/3004	1013/1993	0.81 (0.72–0.90)		1825/3004	1600/1993	0.44 (0.35–0.55)		853/3563	357/2715	1.79 (1.39–2.31)	
Palliative	117	15,939/35,645	14,603/28,707	0.76 (0.74–0.78)		21,845/35,051	20,254/28,158	0.59 (0.55–0.62)		12,828/36,813	8752/29,417	1.20 (1.15–1.26)	
Solid tumours, Palliative setting													
Cancer entities					<0.0001				0.019				0.026
Lung	24	3178/6550	3017/5520	0.75 (0.71–0.80)		4269/6550	4136/5520	0.62 (0.55–0.70)		2224/6754	1727/5504	1.06 (0-99-1.14)	
Breast	19	2022/6479	1750/4917	0.81 (0.76–0.87)		3603/6389	3279/4875	0.64 (0.58–0.70)		1586/6602	873/4976	1.41 (1.21–1.64)	
Colorectal	14	2797/4513	2633/3915	0.80 (0.75–0.86)		3362/4406	2980/3802	0.62 (0.55–0.71)		1849/4751	1422/4092	1.12 (1.01-1.25)	
Renal	11	1443/3933	1595/3781	0.74 (0.68–0.79)		2143/3866	2369/3714	0.60 (0.52–0.70)		1563/4053	1254/3829	1.18 (1.04–1.35)	
Melanoma	10	902/2323	836/1919	0.66 (0.58–0.75)		1287/2262	1290/1857	0.53 (0.42–0.66)		907/2336	620/1910	1.25 (1.03–1.51)	
Sarcoma/GIST	6	296/808	184/477	0.56 (0.43–0.73)		407/808	313/477	0.35 (0.23–0.51)		349/975	185/635	1.21 (0.94–1.56)	
Gastroesophageal	5	1025/1463	776/1003	0.71 (0.64–0.79)		1244/1529	940/1075	0.61 (0.54–0.68)		628/1522	416/1063	1.03 (0.92–1.14)	
Liver	5	936/1681	624/994	0.69 (0.62–0.76)		1011/1681	581/994	0.49 (0.43–0.56)		744/1664	417/983	1.10 (0.94–1.29)	
Prostate	5	1041/3271	843/2067	0.73 (0.66–0.80)		1362/2936	1114/1730	0.55 (0.37–0.82)		904/3251	463/2052	1.23 (0.94–1.59)	
Ovarian	4	662/1522	697/1524	0.87 (0.78–0.97)		956/1522	1052/1524	0.55 (0.41–0.75)		515/1497	323/1509	1.59 (1.18–2.15)	
Head and neck	4	556/768	527/641	0.72 (0.64–0.81)		639/768	595/641	0.70 (0.55–0.89)		514/977	400/838	1.08 (0.99–1.19)	
Drug class					0.02				<0.0001				0.43
Targeted agent	48	4649/13,181	3796/9639	0.74 (0.71–0.78)		6929/12,963	6243/9468	0.48 (0.44–0.52)		4291/13,159	2570/9347	1.25 (1.14–1.37)	
Immune checkpoint inhibitor	30	4312/8560	4270/7117	0.71 (0.68–0.74)		6099/8560	5632/7117	0.72 (0.66–0.78)		3824/9210	2846/7680	1.13 (1.05–1.21)	
Monoclonal antibody	25	4139/7452	4122/7136	0.82 (0.79–0.86)		5298/7411	5381/7095	0.64 (0.59–0.69)		2982/7722	2261/7339	1.26 (1.17–1.36)	
Cytotoxic agent	9	1876/3193	1765/2772	0.77 (0.70–0.85)		2272/2858	1957/2435	0.72 (0.61–0.85)		881/3480	590/3024	1.27 (0.98–1.64)	
Endocrine therapy	4	781/2764	501/1547	0.80 (0.66–0.96)		982/2764	737/1547	0.52 (0.31–0.86)		774/2752	397/1539	1.07 (0.91–1.27)	
Submission type					0.33				0.012				0.29
New active substance	45	5770/14,606	4527/10,392	0.75 (0.71–0.78)		8113/14,281	6938/10,108	0.53 (0.48–0.59)		4716/14,542	2736/10,158	1.25 (1.15–1.37)	
Indication extension	72	10,169/21,039	10,076/18,315	0.76 (0.74–0.79)		13,732/20,770	13,316/18,050	0.62 (0.58–0.66)		8112/22,271	6016/19,259	1.18 (1.11–1.25)	
Treatment type					0.86				0.0019				0.0013
Monotherapy	58	7061/15,790	6070/12,217	0.75 (0.72–0.78)		9185/15,555	8314/11,975	0.53 (0.48–0.58)		5702/16,235	3950/12,284	1.11 (1.04–1.18)	
Combination therapy	59	8878/19,855	8533/16,490	0.77 (0.74–0.79)		12,660/19,496	11,940/16,183	0.64 (0.61–0.68)		7126/20,578	4802/17,133	1.30 (1.22–1.39)	
Treatment line					0.30				0.16				0.13
First line	58	5900/15,546	6256/14,057	0.74 (0.71–0.78)		8431/15,216	9128/13,730	0.59 (0.55–0.64)		5769/16,361	4259/14,639	1.23 (1.15–1.32)	
Second and/or further line	55	9616/19,104	8101/14,057	0.77 (0.74–0.79)		12,742/18,840	10,634/13,835	0.59 (0.54–0.65)		6648/19,471	4323/14,186	1.16 (1.08–1.24)	
Any line	4	423/995	246/593	0.77 (0.65–0.93)		672/995	492/593	0.43 (0.34–0.56)		411/981	170/592	1.53 (1.11–2.11)	
Time of approval					0.043				0.36				0.30
2001–2005	8	1254/2276	1029/1809	0.80 (0.73–0.87)		1018/1874	814/1405	0.60 (0.47–0.76)		677/2274	380/1813	1.36 (1.22–1.51)	
2006–2010	18	2519/5540	2513/5133	0.80 (0.75–0.86)		3606/5499	3587/5092	0.63 (0.56–0.71)		2168/5813	1629/5326	1.25 (1.09–1.44)	
2011–2015	35	4660/10,131	4213/8349	0.78 (0.75–0.81)		6404/10,070	6090/8287	0.54 (0.48–0.60)		3701/10,396	2495/8378	1.24 (1.13–1.37)	
2016–2020	56	7506/17,698	6848/13,416	0.72 (0.69–0.75)		10,817/17,608	9763/13,374	0.60 (0.55–0.65)		6282/18,330	4248/13,900	1.15 (1.08–1.22)	
Marker-based indication					0.55				0.81				0.34
No	70	11,204/23,401	10,013/18,818	0.76 (0.74–0.79)		14,817/22,999	13,412/18,414	0.59 (0.54–0.64)		8624/23,459	5884/18,613	1.18 (1.11–1.25)	
Yes	47	4735/12,244	4590/9889	0.75 (0.71–0.79)		7028/12,052	6842/9744	0.58 (0.54–0.63)		4204/13,354	2868/10,804	1.24 (1.15–1.35)	
Study phase					0.13				0.82				0.66
II	8	572/1202	437/888	0.75 (0.64–0.86)		740/1112	654/846	0.60 (0.50–0.73)		439/1181	281/867	1.25 (1.06–1.47)	
III	109	15,367/34,443	14,166/27,819	0.76 (0.74–0.78)		21,105/33,939	19,600/27,312	0.58 (0.55–0.62)		12,389/35,632	8471/28,550	1.20 (1.14–1.26)	
Control arm					0.30				<0.0001				0.94
Active control	90	11,761/25,994	11,915/23,081	0.76 (0.74–0.79)		16,494/25,574	16,560/22,712	0.64 (0.60–0.67)		9370/27,193	7019/23,808	1.20 (1.14–1.27)	
Placebo control/no treatment	27	4178/9651	2688/5626	0.73 (0.69–0.76)		5351/9477	3694/5446	0.44 (0.39–0.49)		3458/9620	1733/5609	1.21 (1.08–1.35)	
Blinding type					0.84				0.0009				0.13
Open label	62	8337/16,722	8690/15,601	0.76 (0.73–0.79)		10,902/16,285	11,042/15,161	0.64 (0.60–0.69)		6481/17,947	5054/16,389	1.16 (1.09–1.24)	
Double blinded	55	7602/18,923	5913/13,106	0.75 (0.72–0.78)		10,943/18,766	9212/12,997	0.53 (0.49–0.57)		6347/18,866	3698/13,028	1.25 (1.17–1.34)	
Crossover^d^					0.78				<0.001				0.71
Allowed	23	1698/5118	1604/4091	0.75 (0.69–0.83)		2686/5011	2822/3978	0.46 (0.39–0.54)		1671/5301	1120/4210	1.18 (1.04–1.34)	
Not allowed	94	14,241/30,527	12,999/24,616	0.76 (0.74–0.78)		19,159/30,040	17,432/24,180	0.62 (0.58–0.65)		11,157/31,512	7632/25,207	1.21 (1.15–1.27)	

Subgroups with ≥ four trials were included in the analyses. HR = Hazard Ratio. RR = Risk Ratio.CI = confidence interval. GIST = Gastrointestinal Stromal Tumour.

a Includes progression-free survival (n = 113), time to progression (n = 2), and metastasis-free survival (n = 2) in the palliative setting, disease-free survival (n = 10), relapse-free (n = 2), and event-free survival (n = 1) in the curative setting, and progression-free survival (n = 12) in maintenance setting.

b P-values for moderators were obtained using an F test with Knapp-Hartung adjustment.

c Events for serious adverse events refers to the number of patients with at least one serious adverse event.

d Protocol allowed crossover from the control arm to the experimental treatment before primary analysis of the trial.

e RR was calculated as the risk in the experimental arm divided by the risk in the control arm.

In contrast, treatment was associated with an increased relative risk of SAEs by 26% (RR 1.26; 95% CI 1.21–1.32; I^2^= 82.1%). For AEs grade 5 (death), the pooled RR was 0.98 (95% CI 0.91–1.05) (Appendix p 3). There was inconsistent reporting of AEs, with 120 trials reporting AEs grade 3–4. The absolute risk of AEs was higher in experimental arm compared to control across all trials (RD 11.54; 95% CI 8.10–14.98) (Appendix p 4).

Overall, among 93 (38.6%) trials reporting median OS and 146 (60%) reporting median PRTTE (PFS) at approval in both arms, absolute median gains were 2.42 (95% CI 2.11–2.73) months for OS and 3.47 (95% CI 2.89–4.04) months for PRTTE (Appendix p 5). Looking at response rates, in solid tumours, the odds of achieving a response in the treatment arm were 2.46 times compared with control (OR 2.46; 95% CI 2.20–2.74; I^2^= 80.4%) (Appendix p 6).

We then stratified trials by cancer type. For OS, the pooled HRs were similar for solid tumours (HR 0.76 [95% CI 0.74–0.78; I^2^= 23.2%]) and haematological neoplasms (HR 0.75 [95% CI 0.70–0.79; I^2^= 28.8%]). The PRTTE (PFS/DFS) benefits were consistently larger in both as compared to OS benefit (solid tumours: PRTTE HR 0.57 [95% CI 0.54–0.60; I^2^= 86.7%]; haematological neoplasms: PRTTE HR 0.50 [95% CI0.44–0.57; I^2^= 86.3%]). The absolute risk of SAEs was higher in experimental than in control arms in both solid and haematological neoplasms (solid: 33.65% [95% CI 31.69–35.61] vs. 27.15% [95% CI 25.06–29.23]; haematological: 45.97% [95% CI 41.15–50.80] vs. 37.76% [95% CI 33.26–42.27]) (Appendix p 7).

We conducted a subgroup meta-analysis across solid tumour trials in the palliative setting. OS benefit varied significantly across cancer types (p < 0.0001) and drug classes (p = 0.02), with the greatest benefit observed in sarcoma/GIST (HR 0.56 [95% CI 0.43–0.73]) and the least in ovarian cancer (HR 0.87 [95% CI 0.78–0.97]) (Table 2). Across drug classes, immune checkpoint inhibitors showed the greatest OS benefit (HR 0.71 [95% CI 0.68–0.74]), compared with monoclonal antibodies (HR 0.82 [95% CI 0.79–0.86]). PRTTE (PFS) also varied across cancer types (p = 0.019) and drug classes (p < 0.0001), with the greatest benefit observed in sarcoma/GIST (HR 0.35 [95% CI 0.23–0.51]) and with targeted agents (HR 0.48 [95% CI 0.44–0.52]), compared with head and neck cancer (HR 0.70 [95% CI 0.55–0.89]) and cytotoxic agents (HR 0.72 [95% CI 0.61–0.85]). PRTTE effects also differed by comparator type, with greater benefit in placebo-controlled trials (HR 0.44 [95% CI 0.39–0.49]) than in active-controlled trials (HR 0.64 [95% CI 0.60–0.67]; p < 0.0001). The risk of SAEs varied across cancer types (p = 0.026), with the highest RR in ovarian cancer (RR 1.59 [95% CI 1.18–2.15]) and lowest in gastroesophageal cancer (RR 1.03 [95% CI 0.92–1.14]).

**Fig. 3:**
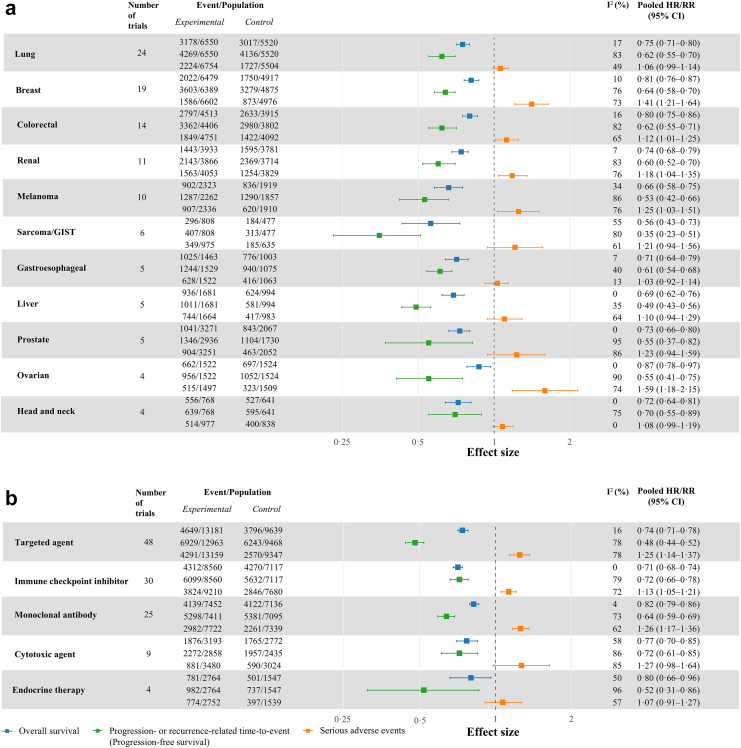
**Treatment outcomes for overall survival, progression- or recurrence-related time-to-event, and serious adverse events of randomised controlled trials supporting solid tumour drug approvals in the palliative setting in Switzerland, 2001–2020.** CI = confidence interval. I^2^ = the degree of heterogeneity across studies. HR = hazard ratio. RR = risk ratio. GIST = gastrointestinal stromal tumour. Forest plots show pooled treatment effects for OS (blue line), PRTTE (progression-free survival) (green line), and SAEs (orange line). The figure highlights differences in the treatment effects between PRTTE and OS across cancer types and drug classes, illustrating that the degree of alignment between progression-based endpoints and survival varies across these subgroups. (a) Across solid tumour cancer types. (b) Across drug classes. Hazard ratios derived from a random-effects model with the restricted maximum likelihood (REML) method. Risk ratios were derived using a random-effects model with the inverse-variance model. Only subgroups with ≥ four trials were included in the analysis. For serious adverse events, events refer to the number of patients with at least one serious adverse event.

Looking at treatment effect differences between OS and PRTTE (PFS), we observed a variation across cancer types and drug classes in the palliative setting (Fig. 3). In some cancer types like ovarian cancer, the difference between pooled HRs of OS and PRTTE was pronounced (OS HR 0.87 vs. PRTTE HR 0.55). In contrast, in cancers like head and neck, pooled HRs for OS and PRTTE were more closely aligned (OS HR 0.72 vs. PRTTE HR 0.70). Across drug classes, while targeted agents showed a larger difference between treatment effect for OS and PRTTE (OS HR 0.74 vs. PRTTE HR 0.48), in immune checkpoint inhibitors the pooled HRs of OS and PRTTE were more comparable (OS HR 0.71 vs. PRTTE HR 0.72). In exploratory analyses, differences between OS and PRTTE varied by line of therapy, differing between first line and later line settings across cancer types in palliative setting (Appendix p 9).

## Discussion

Our study systematically evaluated the clinical evidence supporting cancer drug approvals by Swissmedic between 2001 and 2020, focusing on efficacy and safety outcomes reported at the time of regulatory approval. The use of Swissmedic data offers relevant insights, as its regulatory evidence base and decisions are generally well aligned with those of the EMA and FDA.^28^

In total, 241 RCTs supported the approvals of 102 distinct cancer drugs. At approval, cancer drugs were associated with reduction in the risk of death (24%) and progression or recurrence (45%). These relative benefits translated into modest median absolute survival gains (OS 2.42 months; PRTTE 3.47 months), based on a limited subset of trials with available median OS and PRTTE (PFS) in both arms. However, long-term survival, an important measure to assess clinical benefit of drug interventions, was generally not available at the time of approval. As a result, HRs and median survival at approval may underestimate the ultimate benefit of a therapy in terms of long-term survival. Our findings are consistent with previous studies of cancer drug approvals, which similarly report modest survival improvements at the time of approval and variability in endpoint selection and clinical benefit.^13^^,^^15^ Michaeli et al.^13^ reported pooled HRs of 0.73 for OS and 0.57 for PFS, corresponding to median survival gains of approximately 2–3 months for FDA approvals (2003–2021). Similarly, prior study in Switzerland has shown that 39% of trials supporting approvals meet the ESMO-MCBS threshold for substantial clinical benefit at the time of approval.^29^

In terms of safety, we found that newly approved drugs were associated with an increased risk of SAEs (RD 6.91%) and a higher risk of grade 3–4 (RD 11.54%) compared with control arms, consistent with previous studies.^30^

OS remains underused as a primary endpoint, being employed in less than a third of approvals. However, OS is increasingly included as a key secondary endpoint, formally controlled for multiplicity within hierarchical or graphical testing strategies that preserve the overall type I error rate.^31^ PRTTE endpoints, mostly PFS, remain the most common primary endpoint, reflecting a pragmatic approach to generate earlier efficacy data to support regulatory decisions. However, the validity of these endpoints as surrogates for OS remains uncertain and appears to vary across tumour types, treatment modalities, and lines of therapy.^32^

Across trials, we observed consistently larger effects for PRTTE (PFS/DFS) than for OS. This is consistent with previous studies and suggests that early endpoints may overestimate the true magnitude of survival benefit.^13^^,^^15^^,^^16^^,^^33^ In our analysis, we described the magnitude of this “shrinking effect” between OS and PRTTE endpoints and showed that it varied by cancer type and drug class in the palliative setting. Some tumour types such as head and neck cancer showed relatively consistent treatment effects for OS and PRTTE (PFS). In contrast, others such as ovarian cancer exhibited substantial discrepancies between these endpoints. These differences may arise from variations in disease biology, mechanism of action, and treatment sequencing. Furthermore, our exploratory analyses suggest that the relationship between OS and PRTTE may vary by treatment line.

Targeted therapies tended to show larger effects on early endpoints than on OS, potentially reflecting acquired resistance and subsequent treatment adapted to the mechanism of resistance, whereas immune checkpoint inhibitors demonstrated more concordant effects, possibly due to durable responses in a subset of patients. However, the OS benefit for targeted agents was very similar to that of immune checkpoint inhibitors. These findings underscore the biological and clinical factors that may influence the relationship between early endpoints and long-term outcomes and highlight important considerations when interpreting early evidence.

Taken together, our findings highlight the challenges faced by regulators and clinicians when relying on early efficacy endpoints. The limited availability of primary analysis and long-term OS data, together with variability in the association between PRTTE and OS, suggests that early endpoints should be interpreted cautiously, particularly when extrapolating to long-term survival benefit. While early endpoints can facilitate timely access to new therapies, their use should be accompanied by robust post-approval evidence generation to confirm survival benefit and more comprehensively characterise safety.

While our study offers new insights, limitations must be acknowledged. First, additional outcomes such as quality of life were not included, as analyses of Swissmedic approvals indicate that these data are heterogeneously reported and insufficiently standardised for pooled meta-analysis.^34^ Second, meta-regression analyses were limited to univariate models due to the risk of overfitting given the available data. Third, safety analyses were limited by the lack of individual patient data and primarily relied on SAEs, as reporting of AEs grade 3–4 was available only for a subset of trials. Fourth, substantial heterogeneity was observed across trials for PRTTE, reflecting differences in endpoint definitions, assessment methods, follow-up, and patient populations. Although random-effects models were used, pooled estimates should be interpreted with caution, as they represent average effects across heterogeneous contexts and may not be fully generalisable. In addition, PRTTE and OS endpoints may be influenced by informative censoring,^35^^,^^36^ which could affect treatment-effect estimates, particularly in the absence of individual patient data. We also did not assess the adequacy of comparator arms beyond broad classification, a complex and context-dependent issue that may influence observed effect sizes. Finally, this study includes only trials submitted for regulatory approval and may therefore be subject to publication or submission bias, as studies with unfavourable results are less likely to be submitted. Formal assessment of publication bias was not feasible given the nature of the dataset.

In conclusion, cancer drugs approved by Swissmedic were associated with treatment benefits at the time of approval, but these translated into modest absolute survival gains and were accompanied by increased risk of AEs. Important evidence gaps remain at the time of regulatory approval, including limited availability of median survival gain, long-term OS data, and a frequent reliance on early or intermediate endpoints, whose validity as surrogates varies across clinical settings. These findings highlight the importance of systematic and transparent post-approval monitoring and careful interpretation of early endpoints, taking cancer type and drug class into account to ensure meaningful long-term patient benefit.

## Contributors

AW (Wicki), PR, and U-PR led the study concept and design. SDT provided the list of approvals. PR led the collection and analysis of the data. QL provided statistical expertise. All authors had access to the data and analyses. QL, LR, SDT, NM, JB, NB, AG, MI, SJ, KE, BM, EZ, MZ-P, and AW (Wolfer) accessed and verified the data, and conducted data monitoring and quality assurance. AW (Wicki), PR, U-PR, AW (Wolfer), QL, and LR contributed to the interpretation of the data. AW (Wicki), PR, and U-PR drafted and revised the manuscript. All authors contributed to the review and editing of the manuscript and had final responsibility for the decision to submit for publication.

## Data sharing statement

Due to legal reasons in Switzerland, the data are not publicly available, nor can they be made available beyond the information given in the publication.

## Declaration of interests

AW (Wicki) is the academic supervisor of the Precision Oncology Program, a public-private partnership between the University of Zurich, University Hospital Zurich, and F. Hoffmann-La Roche. AW (Wicki) is also a co-inventor in a patent on the analysis of circulating tumour cells by apheresis, Captaim study (Application PCT/EP2025/052381/WO2025163057A1). U-PR declares travel expenses covered by the organisers for ESMO 2025 as an invited congress speaker and as speaker for The New York Lung Cancer Foundation (NYLCF) annual meeting Lung Cancer: Call-To-Action, March 2026. All other authors declare no competing interests.

## Appendix A Supplementary data

Supplemetary material

PRISMA_checklist
