# Supplementary material for: Efficacy and Safety Evidence Supporting Cancer Drug Approvals in Switzerland (2001–2020): A Meta-Analysis of Pivotal Randomised Controlled Trials
Source: Lancet Reg Health Eur. 2026 May 14;66:101710. doi: 10.1016/j.lanepe.2026.101710 (PMC13196316; doi:10.1016/j.lanepe.2026.101710)
Supplement: Supplemetary material [file mmc1.docx]

**Table of Contents**

[eTable 1 Cancer drugs and corresponding drug classes approved by Swissmedic in Switzerland, 2001-2020 1](#_Toc228183371)

[eTable 2 Meta-analysis of risk ratios for grade 5 adverse events in randomised controlled trials supporting cancer drug approvals in Switzerland, 2001-2020 3](#_Toc228183372)

[eTable 3 Meta-analysis of absolute risks (by treatment group) and risk differences for grade 3**–**4 and grade 5 adverse events 4](#_Toc228183373)

[eTable 4 Median gain of overall survival (OS) and progression- or recurrence-related time-to-event endpoints (PRTTE) in randomised controlled trials supporting cancer drug approvals in Switzerland, 2001-2020 5](#_Toc228183374)

[eTable 5 Meta-analysis of odds ratios for response rate endpoint in randomised controlled trials supporting cancer drug approvals in Switzerland, 2001-2020 6](#_Toc228183375)

[eTable 6 Meta-analysis of absolute risks (by treatment group) and risk differences for serious adverse events 7](#_Toc228183376)

[eTable 7 Comparison of meta-analysis results: Full Cohort vs endpoint-specific matching 8](#_Toc228183377)

[eFigure 1 Meta-analysis of overall survival and progression- or recurrence-related time-to-event outcomes and median survivals by line of therapy in solid tumours in the palliative setting 9](#_Toc227241680)

| **eTable 1 Cancer drugs and corresponding drug classes approved by Swissmedic in Switzerland, 2001-2020** | | |
| --- | --- | --- |
| Drug class | Sub-classes | Generic name |
| Targeted agent | CDK4/6 inhibitors  BTK inhibitors  EGFR inhibitors  ALK inhibitors  PI3K inhibitors  VEGFR inhibitors  BCR-ABL inhibitors  BRAF inhibitors  MEK inhibitors  mTOR inhibitors  PARP inhibitors  FLT3 inhibitors  JAK inhibitors  Proteasome inhibitors  HER2 inhibitors  KIT / PDGFRA inhibitors  BCL-2 inhibitors | abemaciclib  acalabrutinib  afatinib  alectinib  alpelisib  axitinib  bortezomib  bosutinib  brigatinib  cabozantinib  carfilzomib  ceritinib  cobimetinib  crizotinib  dabrafenib  dacomitinib  dasatinib  encorafenib  erlotinib  everolimus  gilteritinib  ibrutinib  idelalisib  imatinib  ixazomib  lapatinib  lenvatinib  lorlatinib  midostaurin  neratinib  nilotinib  niraparib  olaparib  osimertinib  palbociclib  pazopanib  regorafenib  ribociclib  ripretinib  rucaparib  ruxolitinib  sorafenib  sunitinib  talazoparib  temsirolimus  trametinib  tucatinib  vandetanib  vemurafenib  venetoclax |
| Immune checkpoint inhibitor | PD-1 inhibitors  PD-L1 inhibitors  CTLA-4 inhibitors | atezolizumab  avelumab  durvalumab  ipilimumab  nivolumab  pembrolizumab |
| Monoclonal antibody | Anti-CD20 antibodies  Anti-HER2 antibodies  Anti-VEGF/VEGFR antibodies  Anti-EGFR antibodies  Anti-CD38 antibodies  Anti-SLAMF7 antibodies  Anti-CCR4 antibodies  Anti-PDGFRα antibodies  Anti-IL-6 antibodies  Anti-CD52 antibodies | alemtuzumab  bevacizumab  cetuximab  daratumumab  elotuzumab  isatuximab  mogamulizumab  obinutuzumab  ofatumumab  olaratumab  panitumumab  pertuzumab  ramucirumab  rituximab  Siltuximab  trastuzumab |
| Cytotoxic agents | Antimetabolites  Alkylating agents  Antimicrotubule agents  Topoisomerase inhibitors | azacitidine  bendamustine  cabazitaxel  capecitabine  decitabin  docetaxel  doxorubicin  eribulin mesylate  gemcitabine  ixabepilon  oxaliplatin  paclitaxel  pemetrexed  temozolomid  tipiracil  topotecan |
| Antibody drug conjugate | Microtubule inhibitor  DNA-damaging agent | brentuximab  gemtuzumab  inotuzumab  trastuzumab emtasine |
| Endocrine therapy | Androgen receptor pathway inhibitors  Androgen biosynthesis inhibitors  Estrogen receptor pathway inhibitors | abiraterone  apalutamid  darolutamide  enzalutamid  fulvestrant |
| Other | Immunomodulatory  Macrophage activator  Histone deacetylase (HDAC) inhibitors  Oncolytic virus therapy | lenalidomid  mifamurtide  panobinostat  pomalidomid  talimogene laherparepvec |

| **eTable 2 Meta-analysis of risk ratios for grade 5 adverse events in randomised controlled trials supporting cancer drug approvals in Switzerland, 2001-2020** | | | | | |
| --- | --- | --- | --- | --- | --- |
|  | **No. of**  **trials** | **event/total**  **population***  *experimental arm* | **event/total**  **population**  *control arm* | **Risk Ratio**  **(95% CI)** | **I^2^ (%)** |
| **All cancer types** | 198 | 2654/65590 | 2196/56171 | 0·98 (0·91-1·05) | 20·3 |
| **Cancer type** |  |  |  |  |  |
| Solid tumours | 146 | 1806/52604 | 1457/43960 | 0·97 (0·89-1·07) | 23·7 |
| Haematological | 52 | 848/12986 | 739/12211 | 1·01 (0·92-1·11) | 8·4 |
| **Solid tumours, Treatment setting** |  |  |  |  |  |
| Curative setting | 18 | 89/13687 | 82/13989 | 1·20 (0·79-1·84) | 20 |
| Palliative maintenance | 10 | 19/2808 | 33/1951 | 0·71 (0·26-1·90) | 42·2 |
| Palliative setting | 118 | 1698/36109 | 1342/28020 | 0·96 (0·88-1·05) | 19·5 |
| **Solid tumours, Palliative setting** |  |  |  |  |  |
| **Cancer entities** |  |  |  |  |  |
| Lung | 20 | 304/5468 | 251/4233 | 0·98 (0·81-1·20) | 26·8 |
| Breast | 23 | 217/7340 | 172/5537 | 0·88 (0·71-1·08) | 0·0 |
| Colorectal | 14 | 166/4438 | 161/3775 | 0·84 (0·60-1·17) | 51·3 |
| Renal | 9 | 117/3153 | 119/2897 | 0·92 (0·68-1·25) | 5·1 |
| Melanoma | 11 | 124/2628 | 102/2037 | 1·16 (0·80-1·68) | 2·6 |
| Gastroesophageal | 6 | 145/1743 | 126/1287 | 0·80 (0·60-1·08) | 32·4 |
| Sarcoma/GIST | 5 | 52/747 | 33/521 | 1·09 (0·70-1·69) | 7·5 |
| Liver | 5 | 159/1664 | 113/983 | 0·77 (0·54-1·10) | 42·0 |
| Ovarian | 4 | 16/1497 | 16/1509 | 1·02 (0·50-2·06) | 0·0 |
| Head and neck | 4 | 106/977 | 80/838 | 1·01 (0·76-1·33) | 31·9 |
| **Drug class** |  |  |  |  |  |
| Targeted agent | 49 | 704/12972 | 510/9023 | 0·99 (0·86-1·14) | 11·5 |
| Immune checkpoint inhibitor | 27 | 450/8458 | 390/6930 | 0·93 (0·79-1·10) | 24·8 |
| Monoclonal antibody | 26 | 263/7274 | 257/6405 | 0·90 (0·75-1·08) | 0·0 |
| Cytotoxic agent | 9 | 136/3071 | 103/2621 | 1·06 (0·65-1·74) | 68·6 |
| **Submission type** |  |  |  |  |  |
| New active substance | 47 | 608/15516 | 436/10504 | 0·98 (0·84-1·15) | 18·5 |
| Indication extension | 71 | 1090/20593 | 906/17516 | 0·95 (0·85-1·06) | 21·2 |
| **Treatment type** |  |  |  |  |  |
| Monotherapy | 57 | 866/15643 | 622/11574 | 1·00 (0·88-1·13) | 17·8 |
| Combination therapy | 61 | 832/20466 | 720/16446 | 0·92 (0·81-1·04) | 20·5 |
| **Treatment line** |  |  |  |  |  |
| First line | 57 | 569/14706 | 508/12760 | 1·01 (0·87-1·18) | 18·9 |
| Second and/or further line | 57 | 1043/20422 | 798/14668 | 0·91 (0·81-1·02) | 19·0 |
| Any line | 4 | 86/981 | 36/592 | 1·20 (0·82-1·73) | 13·3 |
| **Time of approval** |  |  |  |  |  |
| 2001-2005 | 8 | 60/1802 | 50/1453 | 0·88 (0·61-1·27) | 0·0 |
| 2006-2010 | 18 | 186/5263 | 167/4777 | 0·96 (0·72-1·27) | 38·9 |
| 2011-2015 | 40 | 593/11990 | 446/9154 | 1·10 (0·93-1·29) | 11·6 |
| 2016-2020 | 52 | 859/17054 | 679/12636 | 0·89 (0·78-1·01) | 23·5 |
| **Marker based** |  |  |  |  |  |
| No | 69 | 1155/22771 | 889/17407 | 0·96 (0·85-1·09) | 35·4 |
| Yes | 49 | 543/13338 | 453/10613 | 0·95 (0·84-1·08) | 0·0 |
| **Study phase** |  |  |  |  |  |
| II | 9 | 68/1273 | 55/961 | 0·90 (0·63-1·28) | 0·0 |
| III | 109 | 1630/34836 | 1287/27059 | 0·96 (0·88-1·06) | 21·6 |
| **Control arm** |  |  |  |  |  |
| Active control | 90 | 1178/26211 | 1015/22499 | 0·98 (0·89-1·08) | 7·0 |
| Placebo control/no treatment | 28 | 520/9898 | 327/5521 | 0·89 (0·72-1·13) | 43·0 |
| **Blinding type** |  |  |  |  |  |
| Open label | 62 | 781/17002 | 663/15293 | 1·00 (0·90-1·12) | 3·5 |
| Double blinded | 56 | 917/19107 | 679/12727 | 0·92 (0·79-1·06) | 29·7 |
| Risk ratio calculated as risk in treatment arm divided by risk in control arm  CI=confidence interval. I^2^=the degree of heterogeneity across studies. GIST= gastrointestinal stromal tumour.  *event refers to the number of patients with adverse events grade 5 | | | | | |

| **eTable 3 Meta-analysis of absolute risks (by treatment group) and risk differences for grade 3–4 and grade 5 adverse events** | | | | | | | | |
| --- | --- | --- | --- | --- | --- | --- | --- | --- |
|  | **3–4** | | | | **5** | | | |
|  |  | Experimental  arm | Control  arm | Risk difference, %  (95% CI) |  | Experimental  arm | Control  arm | Risk difference, %  (95% CI) |
|  | **n** | Probability, %  (95% CI, %) | Probability, %  (95% CI, %) |  | **n** | Probability  (95% CI, %) | Probability  (95% CI, %) |  |
| **Cancer types** | **120** | 61·47  (57·83-65·1) | 49·91  (45·9-53·91) | 11·54  (8·10-14·98) | **198** | 4·39  (3·76-5·03) | 4·38  (3·73-5·03) | 0·07  (-0·04-0·18) |
| Solid tumours | **82** | 58·03  (53·93-62·14) | 43·34  (38·82-47·87) | 14·68  (10·53-18·83) | **146** | 3·77  (3·15-4·4) | 3·83  (3·15-4·51) | 0·08  (-0·04-0·19) |
| Haematological | **38** | 68·87  (62·09-75·64) | 64·06  (58·08-70·05) | 4·8  (-0·87-10·47) | **52** | 6·17  (4·49-7·86) | 5·97  (4·41-7·53) | -0·02  (-0·44-0·4) |
| **Solid tumours, Treatment setting** |  |  |  |  |  |  |  |  |
| Curative setting | **11** | 47·45  (33·58-61·31) | 32·76  (18·56-46·97) | 14·72  (0·91-28·53) | **18** | 0·31  (0·17-0·44) | 0·37  (0·16-0·58) | 0·06  (-0·08-0·2) |
| Palliative maintenance | **1** | - | - | - | **10** | 0·38  (0·13-0·63) | 0·67  (0·31-1·03) | -0·24  (-1·11-0·64) |
| Palliative | **70** | 59·99  (55·84-64·14) | 45·4  (40·73-50·06) | 14·57  (10·19-18·96) | **118** | 4·49  (3·77-5·2) | 4·6  (3·79-5·4) | 0·16  (-0·06-0·38) |
| **Solid tumours,**  **Palliative setting** |  |  |  |  |  |  |  |  |
| **Cancer entities** |  |  |  |  |  |  |  |  |
| Lung | **16** | 56·73  (49·13-64·34) | 54·51  (49·01-60·02) | 2·11  (-3·87-8·09) | **20** | 5·52  (3·69-7·34) | 5·24  (3·35-7·13) | 0·08  (-0·84-1·01) |
| Breast | **12** | 64·23  (54·82-73·64) | 29·25  (21·54-36·95) | 34·89  (24·29-45·49) | **23** | 2·42  (1·17-3·66) | 2·71  (1·15-4·27) | 0·08  (-0·27-0·44) |
| Colorectal | **9** | 63·72  (46·09-81·36) | 45·34  (29·43-61·25) | 17·9  (12·05-23·75) | **14** | 3·51  (2·41-4·62) | 4·53  (2·59-6·46) | -0·5  (-1·75-0·76) |
| Renal | **8** | 59·44  (48·2-70·69) | 53·26  (35·82-70·71) | 5·43  (-2·51-13·37) | **9** | 3·41  (1·81-5) | 3·79  (2·14-5·44) | -0·08  (-0·93-0·78) |
| Melanoma | **4** | 57·44  (37·11-77·77) | 46·99  (19·78-74·21) | 10·56  (-3·42-24·54) | **11** | 4·52  (1·79-7·25) | 4·06  (0·21-7·91) | 0·66  (-0·18-1·5) |
| **Drug class** |  |  |  |  |  |  |  |  |
| Targeted agent | **33** | 61·16  (56·17-66·15) | 38·9  (33·27-44·52) | 22·24  (15·26-29·21) | **49** | 4·88  (3·64-6·12) | 4·68  (3·27-6·09) | 0·37  (-0·01-0·75) |
| Immune checkpoint inhibitor | **18** | 58·02  (51·29-64·76) | 57·45  (50·43-64·48) | 0·66  (-4·46-5·79) | **27** | 5·4  (3·81-6·99) | 5·68  (3·94-7·42) | -0·08  (-0·73-0·57) |
| Monoclonal antibody | **12** | 64·75 (  49·44-80·06) | 51·45  (36·33-66·56) | 12·82  (7·99-17·65) | **26** | 3·34  (2·26-4·42) | 3·92  (2·54-5·29) | -0·01  (-0·48-0·45) |
| Cytotoxic agent | **3** | 69·1  (60·06-78·15) | 36·53  (18·4-54·67) | 32·59  (6·66-58·52) | **9** | 4·37  (1·98-6·75) | 4·17  (1·71-6·62) | 0·09  (-1·67-1·85) |
| Endocrine therapy | **3** | 41·41  (24·18-58·64) | 37·29  (15·07-59·52) | 4·15  (-4·08-12·37) | **5** | 3·21  (-0·49-6·91) | 3·52  (-1·02-8·06) | 0·18  (-0·58-0·93) |
| CI=confidence interval.  n refers to the number of trials reporting the toxicity grades.  Risk differences (experimental minus control) were estimated and meta-analysed for each category | | | | | | | | |

| **eTable 4 Median gain of overall survival (OS) and progression- or recurrence-related time-to-event endpoints (PRTTE) in randomised controlled trials supporting cancer drug approvals in Switzerland, 2001-2020** | | | | | | | | | | |
| --- | --- | --- | --- | --- | --- | --- | --- | --- | --- | --- |
|  | **n** | **OS gain (month)**  **(95% CI)** | **I^2^ (%)** | **n** | **OS HR**  **(95% CI)** | **n** | **PRTTE**^*^ **gain (month)**  **(95% CI)** | **I^2^ (%)** | **n** | **PRTTE HR (95% CI)** |
| **All cancer types** | 93 | 2·42 (2·11-2·73) | 35·2 | 194 | 0·76 (0·74-0·77) | 146 | 3·47 (2·89-4·04) | 99·1 | 194 | 0·55 (0·52-0·58) |
| **Cancer type** |  |  |  |  |  |  |  |  |  |  |
| Solid tumours | 84 | 2·36 (2·03-2·68) | 35·7 | 142 | 0·76 (0·74-0·78) | 122 | 3·02 (2·42-3·62) | 99·2 | 142 | 0·57 (0·54-0·60) |
| Haematological | 9 | 3·16 (2·16-4·16) | 3·0 | 52 | 0·75 (0·70-0·79) | 24 | 6·03 (4·6-7·46) | 87·1 | 52 | 0·50 (0·44-0·57) |
| **Solid tumours, Treatment setting** |  |  |  |  |  |  |  |  |  |  |
| Palliative maintenance | 6 | 2·54 (0·56-4·51) | 54·3 | 12 | 0·81 (0·72-0·90) | 9 | 5·97 (2·21-9·72) | 98 | 12 | 0·44 (0·35-0·55) |
| Palliative | 75 | 2·35 (2·01-2·68) | 37·0 | 117 | 0·76 (0·74-0·78) | 110 | 2·72 (2·17-3·26) | 99 | 117 | 0·59 (0·55-0·62) |
| **Solid tumours, Palliative setting** |  |  |  |  |  |  |  |  |  |  |
| **Cancer entities** |  |  |  |  |  |  |  |  |  |  |
| Lung | 12 | 1·82 (1·17-2·47) | 0·0 | 24 | 0·75 (0·71-0·80) | 20 | 2·06 (0·65-3·46) | 97·7 | 24 | 0·62 (0·55-0·70) |
| Breast | 11 | 2·69 (1·66-3·71) | 0·0 | 19 | 0·81 (0·76-0·87) | 16 | 3·56 (2·46-4·66) | 86 | 19 | 0·64 (0·58-0·70) |
| Colorectal | 14 | 2·1 (1·29-2·91) | 65·3 | 14 | 0·80 (0·75-0·86) | 15 | 1·63 (1·06-2·19) | 94·8 | 14 | 0·62 (0·55-0·71) |
| Gastroesophageal | 6 | 1·70 (1·13-2·28) | 16·7 | 5 | 0·71 (0·64-0·79) | 6 | 0·9 (0·31-1·49) | 88·3 | 5 | 0·61 (0·54-0·68) |
| Melanoma | 4 | 5·31 (0·4-10·22) | 84·4 | 10 | 0·66 (0·58-0·75) | 9 | 2·47 (0·81-4·14) | 98·6 | 10 | 0·53 (0·42-0·66) |
| Sarcoma/GIST | 4 | 5·89 (1·9-9·88) | 69·9 | 6 | 0·56 (0·43-0·73) | 6 | 8·39 (0·79-15·98) | 99·1 | 6 | 0·35 (0·23-0·51) |
| Liver | 4 | 2·34 (1·27-3·41) | 0·0 | 5 | 0·69 (0·62-0·76) | 4 | 2·15 (1·17-3·13) | 78·8 | 5 | 0·49 (0·43-0·56) |
| Ovarian | 4 | 2·00 (-0·06-4·07) | 10·6 | 4 | 0·87 (0·78-0·97) | 5 | 3·4 (2·84-3·96) | 6·4 | 4 | 0·55 (0·41-0·75) |
| Head and neck | 4 | 2·84 (1·74-3·95) | 0·0 | 4 | 0·72 (0·64-0·81) | 4 | 0·78 (-0·51-2·07) | 86·7 | 4 | 0·70 (0·55-0·89) |
| **Drug class** |  |  |  |  |  |  |  |  |  |  |
| Targeted agent | 16 | 2·49 (1·82-3·16) | 19·5 | 48 | 0·74 (0·71-0·78) | 38 | 4·87 (3·55-6·2) | 98·4 | 48 | 0·48 (0·44-0·52) |
| Immune checkpoint inhibitor | 18 | 2·34 (1·65-3·04) | 29·0 | 30 | 0·71 (0·68-0·74) | 30 | 1·32 (0·38-2·26) | 98·5 | 30 | 0·72 (0·66-0·78) |
| Monoclonal antibody | 25 | 2·35 (1·62-3·08) | 55·6 | 25 | 0·82 (0·79-0·86) | 27 | 2·22 (1·74-2·69) | 81·4 | 25 | 0·64 (0·59-0·69) |
| Cytotoxic agent | 13 | 1·92 (1·47-2·37) | 0·0 | 9 | 0·77 (0·70-0·85) | 12 | 1·18 (0·7-1·66) | 91·9 | 9 | 0·72 (0·61-0·85) |
| **Submission type** |  |  |  |  |  |  |  |  |  |  |
| New active substance | 24 | 2·73 (2·07-3·4) | 49·0 | 45 | 0·75 (0·71-0·78) | 38 | 3·64 (2·35-4·94) | 99·7 | 45 | 0·53 (0·48-0·59) |
| Indication extension | 51 | 2·21 (1·81-2·61) | 34·1 | 72 | 0·76 (0·74-0·79) | 72 | 2·25 (1·7-2·8) | 96·1 | 72 | 0·62 (0·58-0·66) |
| **Treatment type** |  |  |  |  |  |  |  |  |  |  |
| Monotherapy | 30 | 2·24 (1·73-2·75) | 44·5 | 58 | 0·75 (0·72-0·78) | 49 | 3·11 (1·91-4·3) | 99·2 | 58 | 0·53 (0·48-0·58) |
| Combination therapy | 45 | 2·44 (1·99-2·89) | 30·7 | 59 | 0·77 (0·74-0·79) | 61 | 2·4 (1·94-2·86) | 97·4 | 59 | 0·64 (0·61-0·68) |
| **Treatment line** |  |  |  |  |  |  |  |  |  |  |
| First line | 31 | 2·9 (2·13-3·67) | 45·5 | 58 | 0·74 (0·71-0·78) | 52 | 2·75 (2·13-3·36) | 90·9 | 58 | 0·59 (0·55-0·64) |
| Second and/or further line | 44 | 2·2 (1·84-2·57) | 36·4 | 55 | 0·77 (0·74-0·79) | 58 | 2·64 (1·74-3·55) | 99·6 | 55 | 0·59 (0·54-0·65) |
| **Time of approval** |  |  |  |  |  |  |  |  |  |  |
| 2001-2005 | 8 | 1·97 (1·01-2·92) | 13·9 | 8 | 0·80 (0·73-0·87) | 8 | 2·14 (1·23-3·05) | 80·6 | 8 | 0·60 (0·47-0·76) |
| 2006-2010 | 18 | 2·38 (1·62-3·15) | 53·4 | 18 | 0·80 (0·75-0·86) | 21 | 2·32 (1·62-3·02) | 95 | 18 | 0·63 (0·56-0·71) |
| 2011-2015 | 17 | 2·32 (1·64-3·00) | 35·5 | 35 | 0·78 (0·75-0·81) | 33 | 2·77 (1·82-3·72) | 98·1 | 35 | 0·54 (0·48-0·60) |
| 2016-2020 | 32 | 2·32 (1·84-2·81) | 28·4 | 56 | 0·72 (0·69-0·75) | 48 | 3·07 (1·93-4·22) | 99·1 | 56 | 0·60 (0·55-0·65) |
| **Marker-based** |  |  |  |  |  |  |  |  |  |  |
| No | 50 | 2·01 (1·7-2·32) | 20·4 | 70 | 0·76 (0·74-0·79) | 70 | 2·49 (1·73-3·24) | 99·5 | 70 | 0·59 (0·54-0·64) |
| Yes | 25 | 3·48 (2·6-4·36) | 40·1 | 47 | 0·75 (0·71-0·79) | 40 | 3·11 (2·37-3·85) | 87·9 | 47 | 0·58 (0·54-0·63) |
| **Study phase** |  |  |  |  |  |  |  |  |  |  |
| II | 5 | 4·35 (2·22-6·48) | 0·0 | 8 | 0·75 (0·64-0·86) | 6 | 1·25 (-0·15-2·66) | 77·7 | 8 | 0·60 (0·50-0·73) |
| III | 68 | 2·28 (1·94-2·62) | 37·2 | 109 | 0·76 (0·74-0·78) | 101 | 2·84 (2·25-3·43) | 99·1 | 109 | 0·58 (0·55-0·62) |
| **Control arm** |  |  |  |  |  |  |  |  |  |  |
| Active control | 60 | 2·39 (2·00-2·78) | 25·0 | 90 | 0·76 (0·74-0·79) | 87 | 2·21 (1·76-2·65) | 96·4 | 90 | 0·64 (0·60-0·67) |
| Placebo control/no treatment | 15 | 2·21 (1·54-2·87) | 65·0 | 27 | 0·73 (0·69-0·76) | 23 | 4·53 (2·27-6·8) | 99·8 | 27 | 0·44 (0·39-0·49) |
| **Blinding type** |  |  |  |  |  |  |  |  |  |  |
| Open label | 50 | 2·66 (2·19-3·13) | 38·8 | 62 | 0·76 (0·73-0·79) | 64 | 1·96 (1·46-2·45) | 92·4 | 62 | 0·64 (0·60-0·69) |
| Double blinded | 25 | 1·95 (1·54-2·36) | 20·8 | 55 | 0·75 (0·72-0·78) | 46 | 3·85 (2·66-5·04) | 99·7 | 55 | 0·53 (0·49-0·57) |
| OS=overall survival. PRTTE=progression-related time to event. CI=confidence interval. I^2^=the degree of heterogeneity across studies. HR=hazard ratio. GIST= gastrointestinal stromal tumour.  ^*^PRTTE Includes progression-free survival in all trials.  No. of trials refers to the number of trials pooled for each analysis.  Median gains in OS and PRTTE (treatment minus control) were pooled using Wald approximation-based approach implemented in R package metamedian.  Hazard ratios for OS and PRTTE from the matched dataset are reported to allow comparison with absolute median gains. | | | | | | | | | | |

| **eTable 5 Meta-analysis of odds ratios for response rate endpoint in randomised controlled trials supporting cancer drug approvals in Switzerland, 2001-2020** | | | | | |
| --- | --- | --- | --- | --- | --- |
|  | **No. of**  **trials** | **No. of responders/total**  **population**  *experimental arm* | **No. of**  **responders /total**  **population**  *control arm* | **Odds Ratio**  **(95% CI)** | **I^2^ (%)** |
| **Cancer types** | 202 | 22722/51456 | 14791/44213 | 2·59 (2·35-2·85) | 82·6 |
| Solid tumours | 140 | 12126/36574 | 6786/30079 | 2·46 (2·20-2·74) | 80·4 |
| Haematological | 62 | 10596/14882 | 8005/14134 | 2·88 (2·39-3·46) | 86·1 |
| **Solid tumours, Treatment setting** |  |  |  |  |  |
| Curative setting | 5 | 587/860 | 494/854 | 1·59 (1·30-1·95) | 0·0 |
| Palliative maintenance | 10 | 280/2287 | 88/1742 | 2·48 (1·81-3·38) | 18·8 |
| Palliative | 125 | 11259/33427 | 6204/27483 | 2·51 (2·23-2·82) | 82·0 |
| **Solid tumours, Palliative setting** |  |  |  |  |  |
| **Cancer entities** |  |  |  |  |  |
| Lung | 23 | 2820/5944 | 1793/5163 | 2·10 (1·68-2·63) | 80·8 |
| Breast | 23 | 2642/6841 | 1232/5172 | 2·34 (1·95-2·81) | 76·5 |
| Colorectal | 14 | 1157/4098 | 849/3765 | 2·12 (1·52-2·94) | 84·8 |
| Renal | 12 | 1181/3991 | 523/3787 | 3·37 (2·19-5·18) | 82·2 |
| Melanoma | 11 | 976/2559 | 476/2008 | 3·67 (2·23-6·03) | 87·4 |
| Gastroesophageal | 6 | 363/1641 | 217/1243 | 1·82 (1·48-2·24) | 0·6 |
| Sarcoma/GIST | 6 | 60/965 | 17/629 | 2·98 (1·07-8·26) | 45·9 |
| Liver | 5 | 163/1671 | 31/988 | 3·01 (2·00-4·53) | 0·0 |
| Ovarian | 4 | 619/930 | 468/949 | 2·31 (1·66-3·20) | 61·3 |
| Head and neck | 4 | 216/768 | 140/641 | 1·93 (1·11-3·35) | 73·8 |
| **Drug class** |  |  |  |  |  |
| Targeted agent | 52 | 4150/13053 | 1775/9542 | 3·41 (2·70-4·30) | 80·0 |
| Immune checkpoint inhibitor | 31 | 3173/8815 | 1950/7415 | 1·91 (1·57-2·31) | 81·6 |
| Monoclonal antibody | 29 | 2921/1957 | 1957/7114 | 2·29 (1·90-2·75) | 83·7 |
| Cytotoxic agent | 9 | 610/2625 | 386/2493 | 1·84 (1·38-2·45) | 65·8 |
| **Submission type** |  |  |  |  |  |
| New active substance | 44 | 3660/11837 | 1431/8608 | 3·17 (2·50-4·03) | 81·3 |
| Indication extension | 81 | 7599/21590 | 4773/18875 | 2·21 (1·95-2·51) | 80·7 |
| **Treatment type** |  |  |  |  |  |
| Monotherapy | 59 | 3684/14453 | 1599/11348 | 3·30 (2·61-4·19) | 81·4 |
| Combination therapy | 66 | 7575/18974 | 4605/16135 | 2·13 (1·89-2·40) | 81·0 |
| **Treatment line** |  |  |  |  |  |
| First line | 66 | 7681/16064 | 4671/14375 | 2·46 (2·08-2·90) | 86·2 |
| Second and/or further line | 54 | 3488/16285 | 1507/12421 | 2·57 (2·15-3·05) | 74·5 |
| Any line | 5 | 90/1078 | 26/687 | 3·26 (2·02-5·27) | 0·0 |
| **Time of approval** |  |  |  |  |  |
| 2001-2005 | 7 | 333/1560 | 193/1353 | 1·97 (1·58-2·46) | 38·0 |
| 2006-2010 | 24 | 1926/6255 | 1128/5873 | 2·73 (2·15-3·48) | 84·2 |
| 2011-2015 | 39 | 3045/10215 | 1337/7958 | 3·51 (2·62-4·70) | 83·9 |
| 2016-2020 | 55 | 5955/15397 | 3546/12299 | 2·05 (1·78-2·36) | 78·8 |
| **Marker-based** |  |  |  |  |  |
| No | 73 | 5455/20777 | 3040/17301 | 2·57 (2·18-3·05) | 81·0 |
| Yes | 52 | 5804/12650 | 3164/10182 | 2·45 (2·07-2·90) | 83·5 |
| **Study phase** |  |  |  |  |  |
| II | 9 | 430/1280 | 197/1021 | 2·38 (1·81-3·14) | 35·3 |
| III | 116 | 10829/32147 | 6007/26462 | 2·53 (2·23-2·87) | 83·0 |
| **Control arm** |  |  |  |  |  |
| Active control | 98 | 10244/26491 | 6086/23336 | 2·27 (2·03-2·55) | 82·9 |
| Placebo control/no treatment | 27 | 1015/6936 | 118/4147 | 5·87 (3·66-9·41) | 65·4 |
| **Blinding type** |  |  |  |  |  |
| Open label | 70 | 6467/17192 | 3778/15952 | 2·38 (2·07-2·75) | 81·6 |
| Double blinded | 55 | 4792/16235 | 2426/11531 | 2·80 (2·25-3·47) | 82·7 |
| CI=confidence interval. I^2^=the degree of heterogeneity across studies. GIST= gastrointestinal stromal tumour | | | | | |

| **eTable 6 Meta-analysis of absolute risks (by treatment group) and risk differences for serious adverse events** | | | | | | |
| --- | --- | --- | --- | --- | --- | --- |
|  | **No. of**  **trials** | **Experimental arm** | | **Control arm** | | **Risk Difference, %**  **(95% CI, %)** |
|  |  | **Probability, %**  **(95% CI, %)** | **I^2^ (%)** | **Probability, %**  **(95% CI, %)** | **I^2^ (%)** |  |
| **Cancer types** | 194 | 36·96 (34·89-39·03) | 97 | 29·99 (27·95-32·04) | 97 | 6·91 (5·56-8·25) |
| Solid tumours | 142 | 33·65 (31·69-35·61) | 96 | 27·15 (25·06-29·23) | 97 | 6·55 (5·08-8·01) |
| Haematological | 52 | 45·97 (41·15-50·8) | 97 | 37·76 (33·26-42·27) | 97 | 8·02 (4·94-11·11) |
| **Solid tumours, Treatment setting** |  |  |  |  |  |  |
| Curative setting | 13 | 26·35 (18·84-33·87) | 99 | 15·31 (11·06-19·55) | 97 | 10·96 (3·61-18·31) |
| Palliative maintenance | 12 | 23·49 (18·83-28·16) | 91 | 13·21 (9·29-17·13) | 90 | 10·04 (5·55-14·54) |
| Palliative | 117 | 35·52 (33·48-37·56) | 95 | 29·94 (27·79-32·09) | 95 | 5·62 (4·18-7·07) |
| **Solid tumours, Palliative setting** |  |  |  |  |  |  |
| **Cancer entities** |  |  |  |  |  |  |
| Lung | 24 | 33·21 (29·23-37·2) | 93 | 31·22 (26·75-35·69) | 94 | 2·08 (-0·17-4·33) |
| Breast | 19 | 24·49 (21·31-27·66) | 90 | 17·44 (14·47-20·42) | 88 | 6·93 (4·17-9·69) |
| Colorectal | 14 | 39·6 (35·32-43·89) | 89 | 35·06 (31·43-38·68) | 82 | 4·45 (0·55-8·34) |
| Renal | 11 | 37·57 (31·46-43·69) | 94 | 32·37 (25·5-39·24) | 95 | 5·55 (1·35-9·75) |
| Melanoma | 10 | 40·32 (31·04-49·59) | 96 | 31·73 (25·46-38) | 89 | 8·25 (1·27-15·22) |
| Gastroesophageal | 5 | 41·21 (36·21-46·21) | 75 | 40·17 (33·36-46·98) | 80 | 1·36 (-2·69-5·42) |
| Sarcoma/GIST | 6 | 35·38 (31·3-39·46) | 42 | 29·18 (22·95-35·41) | 65 | 6·52 (-1·13-14·17) |
| Liver | 5 | 43·77 (37·49-50·06) | 85 | 39·85 (30·45-49·24) | 89 | 4·07 (-2·21-10·35) |
| Ovarian | 4 | 33·29 (28·85-37·73) | 67 | 21·38 (14·21-28·54) | 91 | 12·45 (7·65-17·25) |
| Head and neck | 4 | 52·45 (46·29-58·62) | 74 | 48·6 (41·1-56·09) | 79 | 4·19 (-0·42-8·81) |
| **Drug class** |  |  |  |  |  |  |
| Targeted agent | 48 | 32·8 (30·13-35·46) | 91 | 27·1 (23·77-30·43) | 93 | 5·82 (3·18-8·46) |
| Immune checkpoint inhibitor | 30 | 41·51 (37·18-45·84) | 95 | 36·85 (32·91-40·79) | 93 | 4·77 (1·85-7·69) |
| Monoclonal antibody | 25 | 39·18 (36·09-42·27) | 87 | 31·4 (27·88-34·92) | 91 | 7·87 (5·59-10·15) |
| Cytotoxic agent | 9 | 26·07 (18·69-33·45) | 96 | 21·28 (13·53-29·03) | 97 | 4·67 (-0·45-9·8) |
| Endocrine therapy | 4 |  |  |  |  | 1·79 (-2·03-5·61) |
| **Submission type** |  |  |  |  |  |  |
| New active substance | 45 | 34·14 (31·24-37·04) | 93 | 27·71 (24·54-30·88) | 93 | 6·39 (3·96-8·81) |
| Indication extension | 72 | 36·33 (33·58-39·09) | 95 | 31·29 (28·45-34·13) | 95 | 5·16 (3·36-6·97) |
| **Treatment type** |  |  |  |  |  |  |
| Monotherapy | 58 | 35·27 (32·8-37·75) | 91 | 32·06 (29·11-35·01) | 92 | 3·31 (1·12-5·51) |
| Combination therapy | 59 | 35·8 (32·54-39·05) | 96 | 27·91 (24·86-30·95) | 96 | 7·67 (5·91-9·43) |
| **Treatment line** |  |  |  |  |  |  |
| First line | 58 | 35·54 (32·25-38·83) | 95 | 29·21 (26·12-32·31) | 95 | 6·4 (4·26-8·53) |
| Second and/or further line | 55 | 35·06 (32·45-37·68) | 94 | 30·88 (27·73-34·02) | 95 | 4·29 (2·36-6·23) |
| Any line | 4 | 41·89 (38·81-44·98) | 0 | 27·57 (18·13-37·01) | 85 | 14·06 (4·91-23·21) |
| **Time of approval** |  |  |  |  |  |  |
| 2001-2005 | 8 | 32·03 (20·92-43·14) | 97 | 23·28 (14·89-31·66) | 95 | 7·22 (3·89-10·55) |
| 2006-2010 | 18 | 37 (32·89-41·11) | 91 | 31·07 (25·28-36·86) | 96 | 6·41 (2·71-10·11) |
| 2011-2015 | 35 | 36·55 (32·75-40·34) | 94 | 29·11 (25·62-32·59) | 92 | 7·2 (3·96-10·45) |
| 2016-2020 | 56 | 34·95 (32·01-37·89) | 95 | 31·09 (27·86-34·31) | 95 | 4·24 (2·36-6·13) |
| **Marker-based** |  |  |  |  |  |  |
| No | 70 | 37·48 (34·99-39·97) | 94 | 32·26 (29·44-35·07) | 95 | 5·29 (3·36-7·22) |
| Yes | 47 | 32·56 (29·24-35·89) | 95 | 26·46 (23·34-29·58) | 94 | 6·12 (3·95-8·29) |
| **Study phase** |  |  |  |  |  |  |
| II | 8 | 41·86 (31·77-51·95) | 91 | 32·1 (26·28-37·91) | 69 | 9·23 (1·56-16·91) |
| III | 109 | 35·11 (33·05-37·18) | 95 | 29·81 (27·55-32·07) | 95 | 5·46 (3·98-6·93) |
| **Control arm** |  |  |  |  |  |  |
| Active control | 90 | 35·07 (32·6-37·55) | 95 | 29·67 (27·15-32·18) | 95 | 5·44 (3·88-7) |
| Placebo control/no treatment | 27 | 37·06 (33·86-40·26) | 91 | 30·88 (26·8-34·96) | 91 | 6·27 (2·62-9·91) |
| **Blinding type** |  |  |  |  |  |  |
| Open label | 62 | 36·21 (33·42-38·99) | 94 | 31·54 (28·66-34·42) | 94 | 4·71 (2·59-6·83) |
| Double blinded | 55 | 34·76 (31·75-37·77) | 95 | 28·14 (24·97-31·32) | 95 | 6·6 (4·7-8·5) |
| CI=confidence interval. I^2^=the degree of heterogeneity across studies. GIST= gastrointestinal stromal tumour.  Risk differences (experimental minus control) were estimated and meta-analysed for each category. | | | | | | |

| **eTable 7 Comparison of meta-analysis results: Full Cohort vs endpoint-specific matching** | | | | | | |
| --- | --- | --- | --- | --- | --- | --- |
|  | **OS** | | **PRTTE** | | **SAEs** | |
|  | n | HR (95% CI) | n | HR (95% CI) | n | RR (95% CI) |
| Full cohort | 219 | 0·76 (0·74-0·77) | 226 | 0·56 (0·53-0·58) | 213 | 1·25 (1·19-1-32) |
| Matched dataset | 194 | 0·76 (0·74-0·77) | 194 | 0·55 (0·52-0·58) | 194 | 1·26 (1·20-1·32) |
| OS=overall survival. PRTTE=progression- or recurrence-related time to event. SAEs=serious adverse event. HR=hazard ratio. RR=risk ratio. CI=confidence interval  The primary analysis was conducted on a matched dataset of trials reporting OS, PRTTE, and SAEs (n=194). To address potential bias from excluding trials, separate meta-analyses were performed for each endpoint including all trials reporting that outcome; results were consistent with the matched analysis.  n refers to the number of trials reporting the corresponding endpoint. | | | | | | |


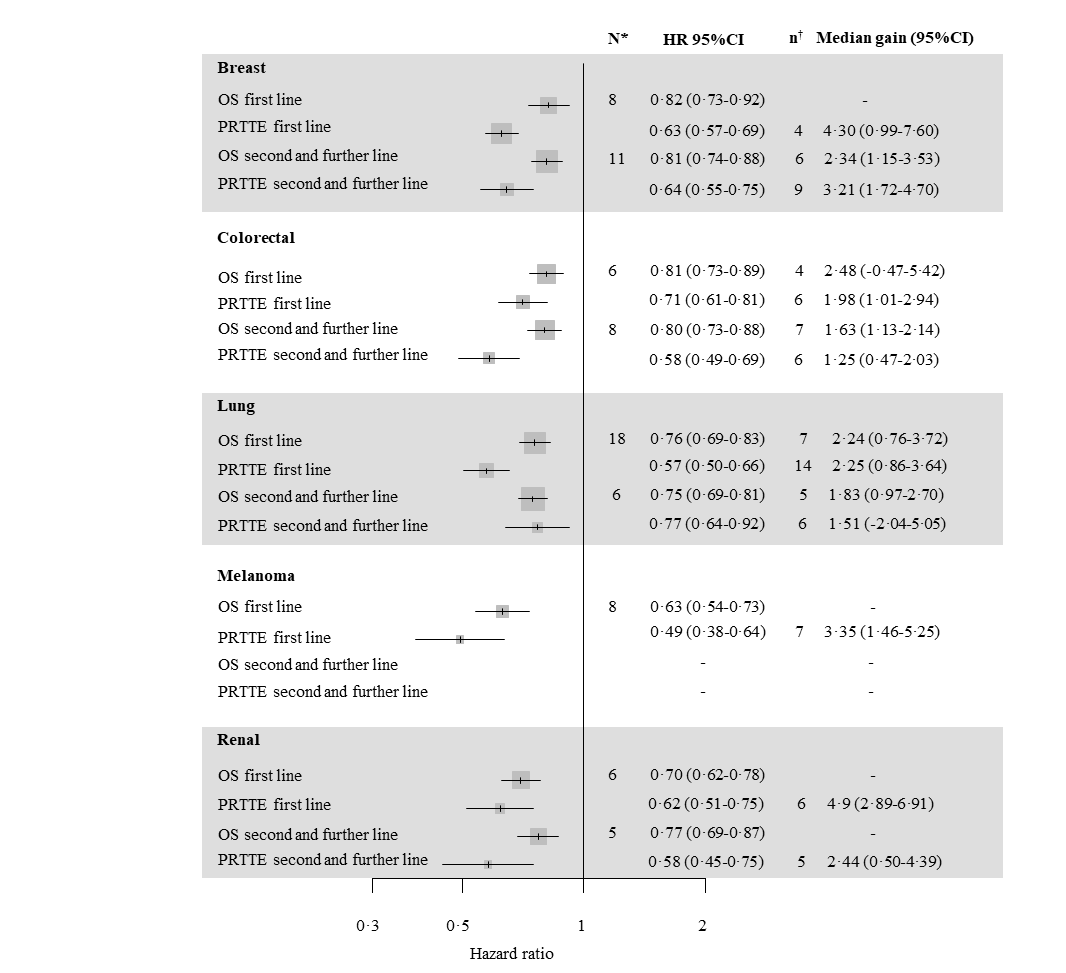


**eFigure 1 Meta-analysis of overall survival and progression- or recurrence-related time-to-event outcomes and median survivals by line of therapy in solid tumours in the palliative setting**

OS=overall survival. PRTTE=progression- or recurrence- related time to event. HR=hazard ratio; CI=confidence interval

PRTTE corresponded to progression-free survival (PFS) in all trials except one breast cancer trial, which reported time to progression (TTP).

Meta-analyses were conducted only for outcomes with ≥4 trials.

Median gains in OS and PRTTE (treatment minus control) were pooled using Wald approximation-based approach implemented in R package *metamedian*.

*N refers to the number of trials reporting OS and PRTTE in each subgroup.

†n refers to the number of trials reporting median OS and PRTTE for both arms in each subgroup.
